# Advancement in Benthic Microbial Fuel Cells toward Sustainable Bioremediation and Renewable Energy Production

**DOI:** 10.3390/ijerph18073811

**Published:** 2021-04-06

**Authors:** Mohammad Faisal Umar, Mohd Rafatullah, Syed Zaghum Abbas, Mohamad Nasir Mohamad Ibrahim, Norli Ismail

**Affiliations:** 1 School of Industrial Technology, Universiti Sains Malaysia, Penang 11800, Malaysia; faisalumar@student.usm.my (M.F.U.); norlii@usm.my (N.I.); 2 Biofuels Institute, School of Environment and Safety Engineering, Jiangsu University, 301 Xuefu Road, Zhenjiang 212013, China; zaghum2009@yahoo.com; 3 School of Chemical Sciences, Universiti Sains Malaysia, Penang 11800, Malaysia; mnm@usm.my

**Keywords:** exoelectrogens, wastewater, benzene, toluene, xylene

## Abstract

Anthropogenic activities are largely responsible for the vast amounts of pollutants such as polycyclic aromatic hydrocarbons, cyanides, phenols, metal derivatives, sulphides, and other chemicals in wastewater. The excess benzene, toluene and xylene (BTX) can cause severe toxicity to living organisms in wastewater. A novel approach to mitigate this problem is the benthic microbial fuel cell (BMFC) setup to produce renewable energy and bio-remediate wastewater aromatic hydrocarbons. Several mechanisms of electrogens have been utilized for the bioremediation of BTX through BMFCs. In the future, BMFCs may be significant for chemical and petrochemical industry wastewater treatment. The distinct factors are considered to evaluate the performance of BMFCs, such as pollutant removal efficiency, power density, and current density, which are discussed by using operating parameters such as, pH, temperature and internal resistance. To further upgrade the BMFC technology, this review summarizes prototype electrode materials, the bioremediation of BTX, and their applications.

## 1. Introduction

In different regions of the world, wastewater rises daily, which is a significant source of pollution in shallow and groundwater [1]. The human immune system is directly affected by water in different ways such as water pollution, degradation, and ecology. Overall, many types of pollutants exist in wastewater, such as organic matter (phenol, pentachlorophenol, nitrobenzene, pyrene, phenanthrene, anthracene) and inorganic matter, which includes nitrogen, phosphorus, ammonia, iron chlorides, nitrate, nitrite and also involves heavy metals [2]. Wastewaters are mostly accumulated in domestic areas from different regions such as laundry wastewater, kitchen utensil wastewater, petrochemical industries and processing plant oil, containing organic compounds and incredibly aromatic hydrocarbons such as benzene, toluene, and xylene (BTX) [3]. Petroleum aromatic hydrocarbons are considered potential water pollutants due to their adverse effects on human beings, including carcinogenic and mutagenic effects [4]. Petrochemical wastewaters are the aggregate composition and the most invasive are hydrocarbons and aromatic hydrocarbons (BTX) [5]. The sewage composition from refinery wastewater includes lubricant and petroleum compounds, which comprise three hydrocarbons such as naphthalene (cyclohexane (C_6_H_12_) and dimethyl cyclopentane (C_7_H_14_)), paraffin (methane (CH_4_), ethane (C_2_H_6_) and propane (C_3_H_8_)), and aromatic compounds (BTX). They are all present in wastewater from petrochemical factories which cause well-known lethal effects. BTX compounds may promote unfavourable health effects in the nervous and respiratory systems. If the desired concentration of BTX can be kept at a minimum level, it will be impossible for the compounds of BTX to have an unfavourable effect on the nervous and respiratory systems of human beings [6]. Amongst the compounds of BTX, benzene is the most hazardous and is extremely cancerous in humans, according to the World Health Organization (WHO 1996) and the International Agency for Research on Cancer (IARC 2012). According to the WHO, a concentration of 1.0 μg/m^3^ of benzene would cause up to six leukaemia cases in a population of 1 million people [7]. In recent years, several techniques have been utilized for the investigation of wastewater before irrigation, such as lagoon ponds, constructed wetlands, and conventional wastewater treatment plants, which involved (coagulation, flocculation, sedimentation, filtration, disinfection, fluoridation, storage and distribution), membrane bioreactor, membrane filtration, precipitation, coagulation–flocculation, adsorption, membrane filtration, and electrochemical treatment technologies. Despite the significance value these techniques provide, the space demands/requirements and huge capital requirements are existing problems dampening their adoption [3,8].

Recently, microbial fuel cells (MFCs) have been considered a substitute source for the bioremediation of vast ranges of BTX, which is non-expensive, and one of the beauties of this method is that microbes from chemical energy generate the electrical energy [9]. The electrical energy produced by any variation of benthic microbial fuel cell (BMFC) depends on the potential difference between non-aerated bio-sediment and aerated water [10]. However, there is a variance between MFCs and BMFCs; in MFCs, synthetic substrate and the non-synthetic substrate are used as fuel for removing toxic substances with renewable energy production; on the other hand, BMFCs utilize natural organic waste substrate as fuel.

**Figure 1:**
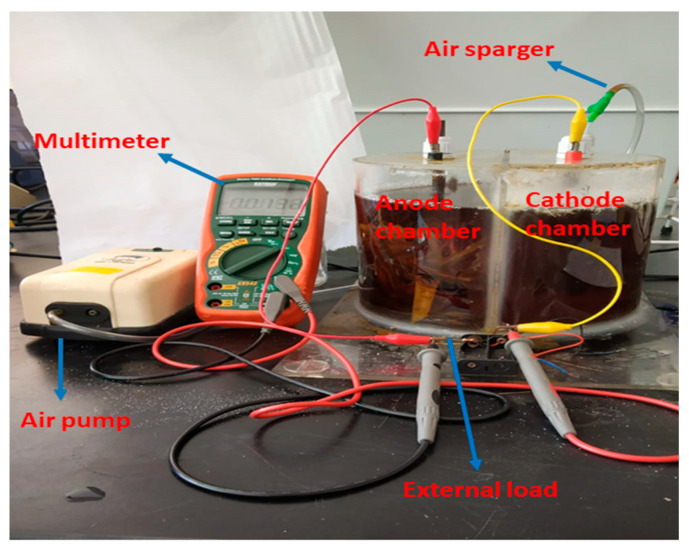
An active benthic microbial fuel cell (BMFC) model.

Actually, natural energy is costly due to existing sustainable energy produced by different energy sources such as solar, wind and hydro-energy, since they rely on the climatic and environmental factors of a particular place. The conclusions that many environmental microorganisms can establish direct electrochemical communication with a solid electrode have led to microbial fuel cell technology [11,12,13,14]. BMFCs have some advantages over the conventional treatment methods because BMFCs have maintainable power generation, they are also progressing at an appropriate fast rate, and a developing scheme illustrated their practical possibility [13]. The prototype of double chamber BMFCs consists of an anode in the non-aerated benthic sugarcane waste and a cathode in the aerated groundwater, which completes the connection for both electrodes of BMFCs from the external circuit. BMFC as a novel creative technology is more effective, less expensive and bio-compatible, which will produce renewable energy as well as the bioremediation of BTX. In previous works, most MFCs consist of an anode and cathode separated by a proton exchange membrane, allowing proton transfer from the anode to the cathode. Previous researchers have used various materials in the construction of the anode and cathode, but it is costly and cannot last for many days. It is also toxic for the growth of microbes, which can hinder the production of electricity. This is why a graphite electrode has been recommended because its unique feature, such as non-toxicity, has long durability and is less expensive. BMFCs can provide an additional advantage in generating electricity, but the primary objective of BMFCs is the bioremediation of BTX pollutants from wastewater. This review summarized advancements in polluted wastewater bioremediation by BMFCs and discussed the approach of renewable electric power generation and an overview of future prospects over different fields. An active BMFC being applied for simultaneous bioelectricity generation and biodegradation of toxicants is shown in Figure 1.

## 2. Configuration of Benthic Microbial Fuel Cell

Potter was the first to harvest electricity via *Escherichia coli* microbes utilizing a platinum electrode in 1911 [15]. Very limited practical advances were attained in the area of microbial fuel cells for the next 55 years. In the past few years, researchers started with electron transfer from bacteria into electrodes using exogenous chemical mediators (natural red, methyl viologen, potassium ferricyanide, thonin, anthraquinone-2, 6-disulfonate, and others); however, these exogenous chemical mediators remain toxic and are unstable [16,17]. The bacteria transfer of an electron to electrodes without any exogenous chemical mediators in the microbial fuel cell field for electricity production was first investigated by Kim et al. [18]. An additional achievement that produced electricity was exhibited through aquatic organic and inorganic matter utilized as substrate in MFCs, and this was examined by Reimers et al. [19]. Electricity produced from organic-rich marine sediments has already been reported in previous work performed through electrochemically active microbes, although a new approach of a special type of microbial fuel cell known as a benthic microbial fuel cell has been employed [20].

**Figure 2:**
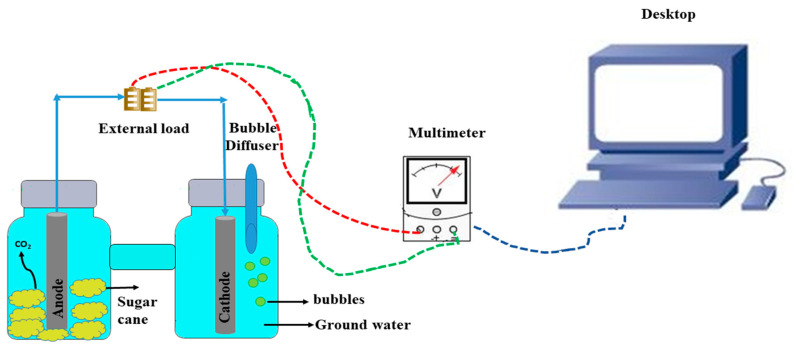
The schematic design and working pathway of a benthic microbial fuel cell with two chambers.

The first kind of BMFCs used carbon fibre and platinum mesh as the anode and cathode, respectively. It was used for the first time in a laboratory discovered by Reimers et al. [19]. After this prototype, a surge of research was financed on BMFCs by modifying the chamber’s electrode design and changing its material to enhance the power generation and its implementation [21]. In recent years, bio-electrochemical reactions have been applied in identifying the conversion of chemical energy into electrical energy in BMFCs. It is one of the new technologies proven through bio-electrochemical reaction to harvest energy between non aerated sediment (plant wastage) substrate and the aerated part overlying groundwater through microbes’ catalytic action. It is a component of the anode that is settled over the wastage plant substrate and connects to the cathode laid down in overlying marine water with a completed cell by an external circuit [22,23]. BMFCs are membrane-less and the boundary is covered by plant wastage, sediment and wastewater. The schematic representation of a double chamber benthic microbial fuel cell is shown in Figure 2.

A string of air cathode in the overlaying water connected with a benthic-integrating bio-anode is the most natural BMFC model. Given the ease and simplicity with which they construct a BMFC setup, the salt concentration and solution conductivity are generally low, but overpotential survives at high conductivity, particularly in freshwater systems [24]. In this case, the anode efficiency decreases because of the decline in the anodic contamination efficiency. The BMFC technique is much more than merely adding sediment or benthic to a polarized electrode. Owing to the large gap of the electrode, there is a loss of a large part of the ohmic BMFCs.

Contrary to laboratory configuration, with the simple operation of correctly separated electrode compartments, a reduction in the electrode gap can be achieved without effort. A BMFC is usually restricted in its proximity to electrodes by the naturally formed spatial separation of oxic and anoxic zones into benthic or sediment [25]. The tubular air cathode design along with the cathodic assembly structure of the fabric suggest that only low-cost fabric would separate the electrodes and the cathode catalytic layer within the tube entered straight toward air to allow the hydrogen oxidation reaction method [26]. Moreover, this setup requires long tubes for air exposure, but the setup of BMFCs cannot operate for a deep-water environment. If they can adapt the cloth cathode without making a setup, then an embedded cathode electrode may be optionally used and under less of the gap of electrodes.

However, the overpotential of BMFCs is a vital influence that confines mass transfer into the benthic region. In particular, the result varies for local pH and has an adverse impact on bacteria physiology by biofilm and benthic under the limited spreading of protons; thus, activation in this crucial way increases overpotential. An attempt to reduce this limit for the anode zone and desire a considerable surface area of carbon fibre can alter the benthic compartment setting in the anode zone [27]. Thus, the result of natural hydraulic flow oxygenation occurs in the anodic area due to strong flow, hence, it should be used carefully. Another thing that should be considered is that there should be mass transfer limitation due to the increase in anode size, thereby creating more interaction area between the anode and benthic. In addition, the increase in the current of BMFCs due to the decrease in internal resistance means a rise in ohmic losses and may result in an unequal proportion of potential distribution through inadequate exploitation of the anode area. Although frequent ohmic losses generally depend on the large surface area of the anode electrode, it was suggested as a potential measure against the creation of a multi-anode network by connecting many smaller anodes with a conventional electronic circuit [28]. Moreover, short-circuited BMFCs (without an additional electrical charge) will further reduce ohms and stimulate anodic reactions. However, the best development and use of these systems could be incredibly site-specific and should be essentially investigated and revealed.

In order to achieve a high anode potential of BMFCs, a working hydrogen oxidation reaction at the anode must occur. Thus, the performance of the BMFC cathode generally requires limited oxygen utilized from heterotrophic microbes and low oxygen solubility in applied configuration. There are various methods to improve the cathode’s oxygen reduction efficiency during MFC experiments involving carbon-based catalysts, metal-based catalysts, metal–carbon hybrids, and metal–nitrogen–carbon complex biocatalysts. The contradictions between the effect of BMFCs’ insignificant zone and the broad sediment region to be handled render it difficult for systematic implementation and constitute another severe consideration for the remediation of BMFCs. It can only affect bioremediation in a vast specific zone by the implantation of the anode in sediment or benthic and other wastage such as spongy medium sand, and usually the electrode surface is several centimetres away. The novel construction of BMFCs exposed to the surroundings through the large surface area anode may be applied through different ways such as a multi-anode and column-type reactors [29]. Recently, a combination of different MFCs was initiated to be considered in practical areas such as the column type of MFCs, which are designed with a graphite granule anode electrode utilized for the bioremediation of adulterated hydrocarbon soil to 70–300 cm, and it was 11–12 times higher than the MFC column radius. However, with the construction of BMFCs, it may be possible to create a novel technology consisting of an anode and cathode chamber. With both electrodes made from graphite separated by a pseudomembrane, it will hopefully help the bioremediation of BTX pollutants [30]. The anode and cathode of BMFCs interact or accept electrons by microbial activity through microbes. Initially, the anode electrode is a terminal electron acceptor from the biological and abiotic process, although the cathode mechanism is to donate electrons available as an acceptor. Generally, oxygen is utilized as an acceptor inside the cathode chamber and it allows for the continuous flow of the anode current.

### 2.1. Anode Chamber

An anode system’s essential characteristics include non-corrosiveness, conductivity, surface area, stability, and biocompatibility, respectively. Therefore, BMFCs’ efficiency will be influenced by the construction method of electrodes and design. Carbon and metal-based anodes are formed from different shapes, such as rods, plates, brush or veil [31]. Thus, metals utilized for anodes, such as silver, nickel, copper, and stainless steel, are commercially available. However, copper conductivity is good, but it is not an appropriate anode material due to its toxicity to microbes. In contrast with anodes made from stainless steel with power densities (12 mW/m^2^) or carbon cloth (CC 880 mW/m^2^), copper can be used for more minutes (2 mW/m^2^). Moreover, stainless steel mesh is an electrode material with good conductivity, but its power density is low as compared to carbon cloth. The stainless steel surface is a problem to stick bacteria due to the smooth surface, although carbon cloth is easy to stick bacteria over the electrode and create a biofilm. Therefore, the electron flows easily through an external circuit from the anode to the cathode by biofilm and produces more power density. In the case of nickel and silver, they are also employed for the anode, but they cannot be utilized for a long time due to corrosion and the unknown time period of microbes. Moreover, stainless steel’s mechanical strength allows it to be easily inserted in the sediment and it also has good conductive power, but it has a relatively small area and it is a liability to the anoxic environment, which makes it less appropriate for BMFC anodes [32,33]. On the other hand, carbon-based materials seem more appealing, even if their electrochemical properties are usually highly diverse in different carbon allotropes and shapes. Rod, sheet, and plate shapes made of carbon-based materials such as graphite have an important role in the BMFC anodes because they are relatively economical in comparison to metal, and they are easy to handle and also have mechanical strength, inertness, commercial availability and they have a fixed surface area. The graphite plate anode has been initially used to design BMFCs to support a continuous power output, but even material plate from graphite is difficult and expensive to be buried into sediment [34]. However, graphite rod has been made to be more easily handled to be inserted into sediment than plate. Anode electrodes of different materials are used to sequence BMFC anodes, but graphite rod electrodes are suitable due to BMFCs’ commercial availability, material strength, and inexpensiveness. 

### 2.2. Cathode Chamber

A reduction reaction occurs in the cathodic system. Electrons are delivered from the anode system toward the cathode system through the external electrical circuit. On the surface of the cathode electrode, electrons and protons combine with oxygen from the air diffuser. Thus, the formation of water in the cathode system occurs with the help of microbes and abiotic catalysis for the reduction reaction of oxygen. There are different types of carbon-based materials such as graphite rods, graphite sheets, and carbon fibres which are utilized for the design of the BMFC cathode and anode. Both the anode and cathode limit the output of MFCs’ power, which means much attention must be given to their design. In addition, platinum materials have shown capability in oxygen reduction reactions as they strongly catalyse cathodic reactions where water is produced from oxygen and protons that migrated from the anode. However, platinum is more expensive than other electrode materials, which also limits its utilization for BMFCs. Although it is significantly superior to graphite and carbon electrodes, it is not sustainable and can be poisonous when used in open bio-cathodes in microbial solutions [35]. Graphite electrodes are a preferred choice over platinum electrodes because they are readily available, cheap, good conductors and do not corrode during reactions.

## 3. Bioremediation Mechanisms of BTX through BMFCs

**Figure 3:**
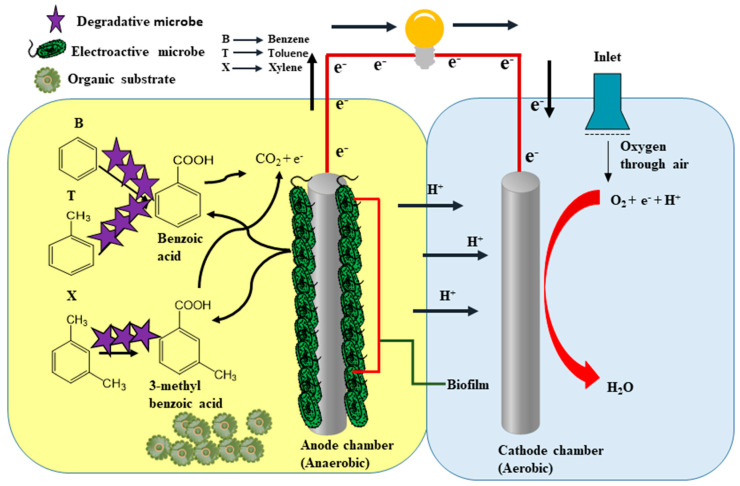
Schematic illustration of general mechanism of benzene, toluene and xylene (BTX) by BMFCs. The bioactivity degradative microbes are pollutants degrading microbes which produced the pollutant intermediates, while electroactive microbes attach to the anode and form biofilm. This biofilm attacks the intermediate to generate CO_2_ and electrons.

Compared to a microbial fuel cell, an anode inserted into the soil and a cathode in the overlying water comprise a benthic microbial fuel cell. In BMFCs, the redox potential difference between the sediment and marine water is responsible for electrons’ movement. The sediment and water edge play an essential role; they work on the microbial fuel cell proton exchange membrane. There are two possible factors in the treatment of BTX pollutant in wastewater by BMFCs. Research has shown that microbial communities are influenced by (i) direct extracellular electron transfer ability and (ii) the use of pollutants as a carbon source [36]. Direct extracellular electron transfer involves exoelectrogens from microbial communities forming biofilms over the electrodes. The anodic biofilm contains electroactive microbes, which are key in the bioremediation process of BTX. Biofilm formation accelerates BTX bioremediation as it encourages electron activation and the breakdown of BTX as a carbon source. These electrons, once activated, migrate to the anode and renewable energy is produced. The diversity of the microbial community, which has both biodegradative and electroactive microbes, makes the mechanism of the bioelectroremediation of BTX unique. These microbes catalyse the anode region, causing the activation of electrons from BTX [37]. These activated electrons move through an external circuit from the anode to the cathode region. The electroactive microbes improve direct extracellular electron efficiency. The biodegradative microbes are responsible for the initial ring cleavage of BTX, while the electroactive microbes are essential in the bioremediation process of BTX. Firstly, benzoic acid is formed under the mediation of carboxylase in a process called carboxylation [38]. Then, benzene and toluene are converted into benzoic acid, but xylene is converted into 3-methylbenzoic acid. This process is as depicted in Figure 3. Benzene is more difficult to convert to benzoic acid through carboxylation when compared to toluene and xylene. This is because benzene may first be converted into thylated or hydroxylated forms before it is converted into benzoic acid. Then, there is the cleavage reaction which converts benzoic acid into smaller acid molecules, CO_2_ and electrons, as shown in Figure 3. 

The MFC technology has been previously researched and reported to have a functional potential to bio-remediate benzene at the anode. Zhang et al. [39] practically stated that the bioremediation of benzene was carried out by a graphite electrode and an electron acceptor from polluted marine-sediment, demonstrating the potential of electrode-based systems for the degradation of aromatic hydrocarbons in anoxic environments. During the concurrent generation of power in 1000 mg of glucose, the author reported that a packaging-type MFC was used and 600 mg of benzene was degraded entirely within 24 h. It was also reported that the degradation of benzene and the production of electricity occurred concurrently with potassium ferricyanide acting as an electron acceptor. The aromatic ring of benzene was possibly triggered in the analysis and cleaved by mono and/or di-oxygenases, suggesting aerobic or micro aerobic conditions as a result of rapid reaction kinetics; benzene can be degraded effectively under oxygen-restricted conditions [40]. 

In another study, benzene was introduced to the system and the production of current was reported. Thirty electrons were said to be theoretically released over the entire degradation of benzene. The production of current depends heavily on the efficiency with which electrons are transported to the anodes as benzene is oxidised [41]. Benzene oxidation is the primary reaction at the anode and the electrons’ release is effectively delivered at the anode, as demonstrated by the anodic and cathodic reaction of MFCs. The degradation efficiency and power density of BTX pollutants have been evaluated by electrochemical reactions such as the anode reaction and cathode reaction [41]. This study’s scope is the bioremediation of BTX pollutants coupled with electricity production using BMFCs [42]. 

## 4. Operating Factors

The study of different parameters that help improve the performance of BMFCs makes this technique more prolific and stable to operate. In this study, synchronization was achieved by BMFCs, which is a biological treatment. BMFCs will operate at lab-scale to enhance the performance of clean energy generation and BTX compound bioremediation by analysing different parameters (pH, temperature, and internal resistance), as briefly discussed below.

### 4.1. Influence of pH on BMFCs’ Performance

BMFCs are remarkably susceptible to external pH in terms of renewable energy generation and contaminant remediation. Whenever external pH changes, many physiological alterations occur, including changes in ionic concentrations, microbial cytosolic pH, proton shuttling, and biofilm formation. In the microbial pathway, pH plays an important role, and it is necessary to examine the pH conditions under which microorganisms can achieve maximum performance [43]. This is a significant aspect in the control of microbial cell metabolism and is also concerned with MFC power generation. The metabolic rate of bacterial cells has been experimented with and optimized at adjacent neutral pH from 6.3 to 7.8. However, when hydrogen stimulation is performed by the degradation of the pollutant, acidic bacteria become active at pH 5.5. The neutral to alkaline pH range decreases the degeneration rate. Consequently, electricity generation is reduced at a low rate of degradation due to the effect of the declining rate of electrons released [44].

The cathode compartment of pH increases from 7–12 with the bacterial community, and thus power production is lowered from 34.7 to 23.8 mW/m^2^, although the 5% CO_2_-air mix generates 100.1 mWm^−2^ [45]. In the beginning, the pH of the MFC did not change, and then it changed with the degradation of waste materials after the reactor operation. Zain et al. [46] stated that the MFCs exhibited small alterations in pH of 1.74% and 1.88% in batch mode and anodic continuous flow, with the pH shift of the ferricyanide reactor at 5.81% over 240 h. In a report by Marashi and Kariminia [47], a single chamber MFC was performed at three different pH levels (5.5, 7.0 and 8.5), with 8.5 being 40% and 66% higher than 5.5 and 7.0 pH. Nitrogen compound denitrification increases the anodic chamber alkalinity, as both biofilm electrodes grow, thus causing maximum power output at pH 9 only. Microalgae control the pH change in the cathodic chamber in BMFCs and simply grow in the cathode chamber at an optimum pH range of 7 to 9. Some authors, including Reimers et al. [19] and Tender et al. [48], found the maximum power density of 10 and 16 mW/m^2^, respectively, for aqueous cathode oxygen limited in the submersed electrode oxygen—consequently, this was mainly limited by the reaction of the oxygen reduction to the aqueous cathode due to the poor kinetics of the neutral pH reaction. For that reason, some researchers have proposed the use of an aerated BMFC cathode with 3.7 times the oxygen stream of an aerated cathode. 

If the power density is dropped due to the slow electron release rate, there will be improved methane production with neutral pH of (6.8–7.2). The activity of acidic bacteria and methanogenic bacteria is reduced with a higher pH value of 8.5, and electrons are also released to contribute to the substrate oxidation process. Therefore, higher pH values (alkaline condition) are appropriate for the development of electrogenic bacteria in BMFCs [49].

### 4.2. Effect of Temperature on BMFCs Performance

The microbial effects in benthic microbial fuel cells as a special type of MFCs are essential for temperature as they are in a simple fuel cell since the bulk of the electrogenic populations are active at 20 °C to 35 °C. Temperature changes also influence the performance of BMFCs. They can affect mass relocation (sediment or benthic conductivity and overlying water, mass transfer coefficient and activation energy), thermodynamics, BMFCs kinetics and the distribution and composition of microbial anodic and cathodic electrodes as each microbial culture has its peak of functioning temperature [50]. The optimal growth rate of active microorganisms and biofilm development also enables optimum pH to be regulated by BMFCs, which is achieved with a varying temperature of about 25 °C to 30 °C. At higher temperatures, there is established biofilm formation and BMFC activity, and thus better performance over a short period, and the initial temperature has been particularly significant in biofilm development. The peak exoelectrogens activity of a biofilm was recorded at a temperature range between 30 °C and 45 °C, and this temperature range is also acceptable for maximum BMFC output. The maximum power density of 894.3 mW/m^2^ that has been reported is found at 30 °C, but in different studies, contaminant extraction and power generation have improved as the temperature increases [51]. The development of metabolic channels will contribute to the augmentation in energy production and contaminant removal and the decline in ohms due to the augmented conductivity of sediments or benthic with surface water. Ohmic resistance response with temperature level is inversely proportional, which means that as the temperature increases, ohmic resistance is decreased. At low temperature, the oxidation rate of organic matter is prolonged. However, power generation extends to an absolute limit with an enhanced temperature, and the bacteria present in the anode biofilm are as follows: *Balobacter* (20 °C) *Zoogloea* and *Simplicispira* (10 °C). Higher operating temperature is useful for removing a more robust chemical oxygen demand [52].

### 4.3. Internal Resistance

The internal resistance of BMFCs depends on the external resistance of the electrolyte and the distance between electrodes. The power generation affected is the vacant space between the electrodes and by the movement of electrons and protons between chambers. In addition, both electrodes will require being closer together as soon as the internal resistance is reduced at the optimum performance of BMFCs. The internal resistance can be minimized by positioning the electrodes close to each other to reduce ohmic losses [53]. It is stated that energy production will be diminished if electrodes are extremely separated as the introduction of oxygen in cathode regions leads to electron losses from anodic sediment or benthic. Aside from a steady supply of oxygen in the bioreactor, the proximity of the electrodes to each other affects the resistance, which in turn affects the voltage produced. This aligns with Ohms’ law that states that voltage decreases when resistance is reduced. Nonetheless, the nearest distance between the electrodes is required in anaerobic BMFCs, since the anode requires anaerobic fermentation. Thus, to enhance the rate of electricity acceptance in all BMFCs and fewer activation losses, it is desired to optimize the distance between electrodes [54].

## 5. Bio-Electron Mechanism Pathways

**Figure 4:**
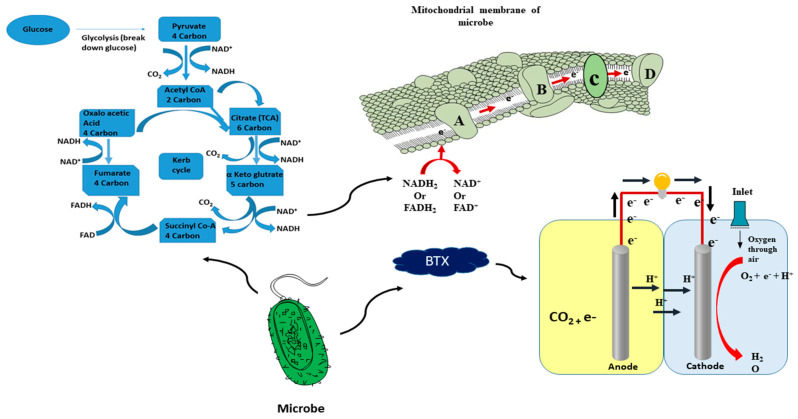
Glucose metabolic pathway and mechanism inside cell membrane of microbes through proteins from electron carriers (NADH and FADH) toward electrode: Proteins (A; NADH dehydrogenase, B; ubiquinone, C; coenzyme, D; cytochromes).

There are different microbes (fungi, yeast, algae, bacteria and even protozoa) in living organisms that can inhabit an extensive variety of organic compounds such as glucose, sugar, proteins, and carbohydrates of equally carbon and energy origin. In the Krebs cycle, organic compounds manage to work as electron donors, although adenosine triphosphate (ATP) particles are like energy transporters prior to cyclic chain reactions. In the glycolytic cycle (break down of glucose), acetyl CoA is formed from assembled monomers of dissimilar organic molecules (carbohydrates, proteins and lipids break) [55]. The CoA by beta oxidation of fatty acid primarily forms citrate. On single complete oscillation throughout the tricarboxylic acid cycle (citric acid cycle or Krebs cycle) forms three equal nicotinamide adenine dinucleotides (NADH) that utilized electrons exporter from the reduction of three equal nicotinamide adenine dinucleotides (NAD^+^), and one flavin adenine dinucleotide (FADH_2_) also utilized electrons exporter from the reduction of one flavin adenine dinucleotide. The carbon dioxide also frees its cycle and the whole mechanism is accomplished in the cell membrane of microbes. These electron transporters or carriers (NADH and FADH) involved in electron relocation toward the electron transport chain produce ATP compound, which acts as an energy carrier. These different microbes carry out a respiratory reaction that occurs in distinct cells with separate cell membranes (build up outer cell membrane, inner cell membrane, and periplasm); for example, bacteria is a prokaryotic cell whose reaction occurs in the cell membrane. However, in yeast, algae and others which contain eukaryotic cells, the electron transport reaction occurs in the inner mitochondrial membrane, but this is absent in prokaryotic cells. The electron transport chain works under four intermediate precursors of enzymes: NADH dehydrogenase, ubiquinone, coenzyme Q, and cytochromes, respectively [56]. The electrons are transferred through the electron transport components (dehydrogenase, quinones, iron sulphur proteins, b-type cytochromes) by enzyme precursors. Finally, electrons are passed away from the final acceptor, and the proton is released by pump proton combined with a final acceptor of oxygen to form water. In an anodic chamber, microbes can be used instead of an electrode as a final acceptor of electrons (terminal acceptor). The electron transport chain (ETC) is a collection of embedded electron carriers inside the inner membrane of microbes, as shown in Figure 4.

**Figure 5:**
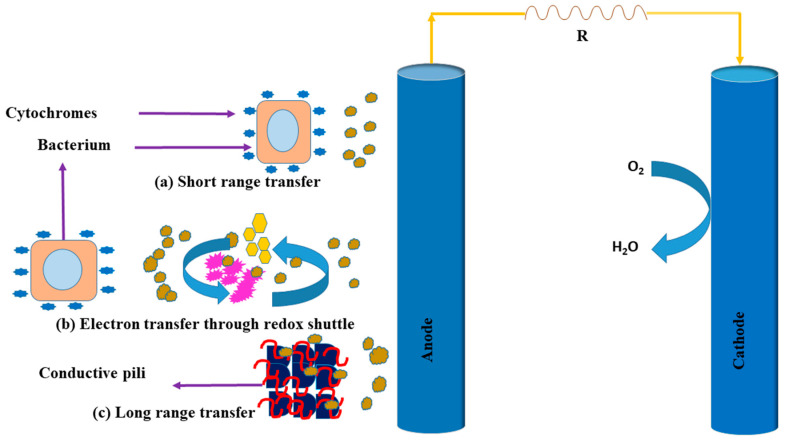
Bio-generation mechanism of electrons through microbes to electrodes: (**a**) short range electron transfer via cytochromes, (**b**) electron transfer via redox shuttles, (**c**) long range transfer via conductive pili, and (R) Resistance.

However, modes of electron transfer toward the electrode from exoelectrogens in MFCs with different redox mediators (neutral red, methyl viologen, etc.) used in prior studies have been reported. These mediators are exploited for the transfer of electrons from inside the cell toward the electrode, and different exoelectrogens have the capability to transfer electrons from the cell to the electrode. The production of electricity in MFCs by many capable exoelectrogens through the path of electron transfer has been used to reveal different mechanisms for transporting electrodes from within a bacterial cell in some bacteria, such as: *Thermincola potens, Geobacter sulfurreducens, Shewanella oneidensis, Geothrix fermentans, Pseudomonas aeruginosa, Thermincola carboxydophila, Shewanella putrefaciens,* and *Escherichia coli* [57,58]. There are different mechanisms involved in the transfer of electrons from exoelectrogens into the electrode, but these mechanisms do not require an artificial electron shuttle short-range electron transfer via redox-active proteins such as cytochromes present on the outer surface of the bacterial cell membrane; electron transport via microbially secreted soluble electron shuttles, long-range electron transfer through conductive pili, as shown in Figure 5.

### 5.1. Electron Shuttling to Electrodes

Diverse microorganisms have different exoelectrogens, and Gram positive and Gram negative bacteria such as *Geobacter fermentans, Shewanella oneidensis, Pseudomonas aeruginosa,* and *Lactococcus lactis* are capable of stimulating electron transfer into the electrode by self-producing electron shuttles. The *Geobacteraceae* are the most prevailing family over the electrode which was embedded into the sediment or benthic, while that embedded at the anode was not connected to the cathode due to the related enhancement. The freshwater benthic where the anode is embedded is the most prominent for the *Geobacter* species, while the *Desulfuromonas* species is the most prominent embedded marine sediment at the anode since these bacteria approach higher salinity [59].

Bond et al. [60] introduced the conception of electron transmission from microorganisms by self-produced soluble electron shuttles for the first time. They have also been implicated in reducing Fe (III) oxides through the liberated soluble electron shuttles of *Geobacter fermentans*. There are two kinds of soluble electron shuttles that are liberated with altered redox potential, although *Geobacter fermentans* grew together with Fe (III) oxides but without a fumarate electron acceptor. The first kind of soluble electron shuttle is riboflavin with an electrode potential of 0.2 V, the other case involves an unknown species with an electrode potential of 0.3 V. However, other species such as *Shewanella* spp. have the capability to augment the transfer of electrons into electrodes and reduce poor crystalline Fe (III) oxides by producing riboflavin and flavin nucleotides as extracellular soluble electron shuttles. The direct electron transfer was possible only by artificially poising the electrodes at greater reduction potentials associated with typical BMFCs, leading to a faster rate of electron transfer. Whenever electron transfer frequency decreases by nearly 60–70% from microbes to electrode, it means that the electron carrier riboflavin has disappeared through the biofilms. In microbes of *Shewanella oneidensis* MR-1, which accelerate extracellular electron transfer, the author reported that the surface area of the carbon cloth electrode acts as cytochrome-bound cofactors from endogenous flavins [61]. The author also reported that different types of electrodes made up of separated materials can impact in different ways, such as the interaction of flavins over the cytochrome electrode surface and microbial action acting as an oxidizing as well as reducing agent. Although flavin adenine dinucleotide acts as an electron carrier to facilitate electron transfer from the external membrane, it still compacts through c-type cytochrome in *Shewanella oneidensis*. This electron carrier transports electrons nearly by 70% from the cell of microbes into the electrode [62]. It also accelerates extracellular electron relocation and controls the path of electrons in *Shewanella oneidensis* MR-1. The multiprotein complexes, which exist in microbes of *Shewanella oneidensis* on the inner membrane and outer membrane, such as MR-1, decaheme Cym A, MtrA (SO4360, MtrD and DmsE), MtrB (SO4359, MtrE and DmsF), OmcA, and MtrC (OmcA and MtrF), all help in the excitation of electrons from the periplasm toward the exterior surface of the membrane. To stimulate the excitation of extracellular electrons in *Shewanella oneidensis* MR-1, Mtr/OmcA is enhanced by flavin. MtrE, MtrF, and MtrD are assumed to make an outer-membrane spanning complex, where MtrFDE is identical to MtrCAB. The DMSO and porin cytochrome DmsEF both necessitate the reduction through the sub unit of DmsAB outside the electron transfer cell. The function of the porin cytochrome complex SO4359_60 and its associated subunit, SO4361_62, is yet to be examined [63]. In *Shewanella oneidensis* MR-1, the cytochrome MTrf present is mobile and forms a strong bond with gold, peptide aromatic compounds and heme groups, and their electron transfer from outside the cell is post-haste as investigated by Wei et al. [41]. One breakthrough by Pirbadian et al. [64] is that the author has to examine if MTrf has greater capacity for the extracellular electron transmission in the recombinant genome as related to the monolayer of Mtrf over flat gold substrate.

### 5.2. Short-Range Electron Conduction via Cytochromes

The exoelectrogens help transfer electrons from inside the microbes’ cells toward the electrode surface as mediator electrons through a direct electron transfer path. However, the evaluation of the *Geobacter sulfurreducens* species’ mechanism with direct electron transfer has been broadly studied previously. *Geobacter sulfurreducens* contains enzyme for central metabolism such that anaerobic cells can oxidize carbon, which can be completely converted into carbon dioxide and water and can transfer electrons to very diverse electron acceptors which include metal ions, elemental sulfur and fumarate. In previous work, the *Geobacter sulfurreducens* employed other means for the transfer of electrons to have direct contact with extracellular electron acceptors such as Fe_2_O_3_, where electrons are generated inside the cytoplasm from central metabolism because they do not use a stuffing box to reduce the electrodes. Several kinds of c-type cytochrome genes are generated from the outer part and contain a central heme group in the *Geobacter sulfurreducens* [65]. The c-type cytochromes remain in electrical communication using the anode, as revealed by several studies on electric current producing biofilms of *Geobacter sulfurreducens*. In some places, c-type cytochromes are placed on close electrode surface for direct extracellular electron transfer; therefore, the mechanism is that there is an electrochemical reaction between the electrode surfaces and microbe cells within contact of the electrode [66].

A contrast study of gene conversion in the cell of *Geobacter sulfurreducens* was carried out on different electrodes and different electron acceptors such as cell development on a graphite electrode and cell growth on fumarate. Different cytochrome types exist in current-harvesting circumstances such as OmcS, OmcT, OmcB, and OmcE, but all of them are not abundant including OmcZ for microarray investigation. Although OmcZ is of two arrangements, one arrangement is short (OmcZs) and the other is long (OmcZL), with different molecular masses of 30 and 50 kDa, respectively, and another that repels water [67]. The OmcZ occupies an extensive spectrum from the redox potential (−60 to −420 mV) of eight heme groups. 

Moreover, it plays a crucial role in developing biofilm on the electrode and transmitting an electron from cells to electrodes. The formation of biofilm by *Geobacter sulfurreducens* developed on the graphite electrode with fumarate was due to the high production of OmcZ on behalf of the immune gold studied. In addition to this, OmcF gene deletion reduces current production because it is not involved in the mechanism of exoelectrogens, but the transcription gene requires current production within the BMFCs [68]. The multicopper proteins, OmpB and OmpC, have been examined to be present in *Geobacter sulfurreducens*, and the outer membrane also has a c-type cytochrome. However, these multicopper proteins are of two domains: one is Fe (III) binding site and the other is fibronectin type (III), which also plays a significant role in the reduction of Fe (III) oxide. Nowadays, as already reported, the c-type cytochrome of *Thermicola potens* has the ability to transfer electrons from some Gram positive bacteria to the electrode [69]. 

### 5.3. Electron Conduction through Conductive Pili

Current is produced as the biofilm becomes conductive due to a complex network of pili formed by microorganisms, which act as intermediates for the long-distance transfer of electrons. However, *Shewanella* species and *Geobacter* species are known as exoelectrogens and they fabricate the pili, which mutually produces the conductive pili. They are also responsible for electricity production, but for the first time, biofilm conduction was revealed in *Geobacter sulfurreducens* [70,71]. A huge amount of pili have a metallic property involved in the conductivity of biofilm for *Geobacter sulfurreducens.* The encoded gene *PilA* with a length, width and molecular weight of 10–20 μm, 3–5 nm, and 7- to 20-kDa, respectively, is the arrangement from protein type IV pili108 [72]. It behaves like a metal with electrical conductivity, and other characteristics like that of metal are as follows: (i) its conductivity increases with a decrease in temperature, as shown with metals, (ii) the *PilA* protein of *Geobacter sulfurreducens* that contains an α-helix and N-terminal also contains a chain of amino acids (Trp, Met, Phe, His, and Tyr) that is attached to another side of the C-terminal, which provides π–π interaction in the form of metal, and (iii) it shoots up the pili conductivity that occurs together with the polyaniline and protons [72].

The fully illustrated pili of *Geobacter metallireducens* indicate the existence of insoluble electron acceptors such as Fe (III) and Mn (IV), although a soluble electron acceptor is not existent in Fe(III) citrate. These conclusions were made considering the following: (i) the *Geobacter sulfurreducens* pili work as microbial nanowires used for the long-range electro transfer into Fe (III) oxides due to the dense biofilm created on the anode electrode of *Geobacter sulfurreducens* in comparison with other microbes [73]. (ii) The nanoparticle of Fe (III) oxide has a strong linkage with the conductive pili. (iii) The *PilA* gene is very important to gain electrons for iron oxide and to protect electron transfer. All these factors give good exploitation, yet *Geobacter* species provided the maximum current with respect to area in comparison to different species [74].

An exoelectrogen that has been studied for microbial fuel cells so far beyond the *Geobacteraceae* family is *Shewanella oneidensis.* Naggar et al. [75] confirmed that *Shewanella oneidensis* MR-1 nanowires are conductive in nature by employing the conducting probe atomic force microscopy technique. In addition, their research has shown the nonconductive nanowire of mutants in genes deficient for c-type decaheme cytochromes, MtrC and OmcA. Further studies demonstrate that p-type, tunable electronic behaviour in field-effect mobility is exhibited in the electronic transport of *Shewanella oneidensis* MR-1 nanowires. The deletion of the pilin genes (mshA-D) in an assisted effort illustrates the extracellular electron transport performance of the intracellular and membrane-bound Msh biogenesis complex in *Shewanella oneidensis* MR-1. In an altered view, the extracellular electron transport capabilities of the structural mannose sensitive hemagglutinin (MSh) protein showed 20% fewer currents in comparison to the control strain. An extracellular charge transfer multistep hopping process in *Shewanella oneidensis* MR-1 biofilms was suggested, proposing the linkage of redox compounds at less than 1-nm distance, developing an extracellular appendages chain responsible for electron jumping or tunnelling [76,77]. However, cytochromes’ actual organization and exact function in electron transmission mechanisms in *Shewanella oneidensis* MR-1 nanowires are not even revealed. 

## 6. BMFCs Employment

The main use of this new technology is to produce renewable energy through benthic microbial fuel cells. Some specific uses of BMFCs are the treatment of wastewater, bioenergy, and biosensors, as follows.

### 6.1. Treatment of Wastewater

In 1990, the microbial fuel cell was first considered as a new technique for the treatment of wastewater [78]. Nowadays, authors have reported several ways of treating municipal, agricultural and industrial wastewater via bioremediation through a great new technology called the benthic microbial fuel cell [79]. High operational sustainability and cheap equipment costs are valuable requirements for a satisfactory treating operation. In addition, MFCs are placed in parallel to control distant areas in combination with other wastewater treatment systems. This was a huge challenge in analogy to researchers, raising the power density even though power density production still has efficiency in the approved standard of ±60%. Table 1 shows the removal efficiency of the various operating conditions by utilizing benthic microbial fuel cells. Studies on BMFCs have been achieved by analysing power generation assessed by cyclic voltammetry, electrochemical impedance spectroscopy, and polarized curves. Moreover, 50% of organic matter is removed from marine sediment obtained from aliphatic compounds at a 10 cm anode depth with a distance between electrodes of 10 cm at an internal resistance of 80 Ω along the current density and power density at 929.7 ± 9.5 mA/m^2^ and 109.6 ± 7.5 mW/m^2^, respectively, with the help of steel mesh electrodes. The author also stated that BMFCs can be constructed for energy utilization and performance, which has a good benefit for the coastal inhabitants, but configuration is suitably well employed at a 10 cm anode depth and 100 cm distance between electrodes with an internal resistance of 110.5 Ω [80]. MFCs and membrane-less MFCs in continuous flow and separate compartments are favourable for wastewater treatment due to concerns with increasing these types of operations. The constructed wetlands developed as MFCs are a successful design approach for enhancing the treatment of domestic wastewater. For 14 weeks, a researcher has developed four lab-scale setups with a membrane-less microbial fuel cell and loaded batch mode with domestic wastewater, and the results showed 220 ohm as the best condition for the highest efficiency of MFCs [81]. 

### 6.2. Bioenergy

The bio-production of electrical energy depends on the electric potential difference between seawater oxygen and sediments without oxygen. One application of BMFCs is the production of energy in the form of easily accessible electrical power. The author has reported that reduced current and power production has been greatly limited in applications driving oceanographic marine instruments. Inside this work, the cerium is coated on the electrode by the electrochemical method, and this novel electrode was applied in the power generation of BMFCs. In many different ways, reactors were set up with combination electrodes. The reactor with coated cerium metal over the anode electrode and platinum coated over the cathode electrode has 5.5 times higher power generation of 63.81 mW/m^2^ than without a coated electrode [32]. Another previous work was also carried out on a modified electrode to increase power generation by the conducting polymer coated over metal oxide [99,100]. The author performed an electrochemical process using polypyrrole (ppy) coated on different metal oxides such as MnO_2_, Fe_2_O_3_ and MnO_2_-Fe_2_O_3_, and then evaluated the surface of electrodes in a benthic microbial fuel cell. Among these modified coated electrodes, ppy-Fe_2_O_3_ has a higher power density (170 mW/m^2^) than other modified electrodes such as ppy-MnO_2_ (90.54 mW/m^2^), ppy-MnO_2_-Fe_2_O_3_ (117.29 mW/m^2^) and without a modified carbon felt fibre electrode (69.19 mW/m^2^). The resultant nanocomposite coated electrodes were used in BMFCs for the treatment of marine sediment and sea water.

Yu et al. [101] designed bioelectrochemical systems (BES) for harvesting energy by wiring-up living cells with abiotic conductive surfaces. The main challenge is the hindrance of the high interfacial area and close contact for material and cell engineering. They proposed a new concept of a single cell electron collector, which is built in situ with an interconnected intact conductive layer on and across the individual cell membrane. Thus, this single-cell electron collector provides a superior method for wired abiotic surface-level living cells and introduces new dimensions for abiotic/biotic interface engineering.

### 6.3. Biosensors 

The BMFCs also applied robust wireless biosensors employed to estimate tidal patterns, temperatures, the migration patterns of marine animals, the existence of marine microbes, salinity, toxins from anthropogenic sources, moisture, pH, dissolved oxygen, and biological oxygen levels. The utilization of electrical energy is clarified for three requirements from the BMFCs’ power management: (i) low voltage should be converted into high voltage as clarified from the biosensor because, initially, the currents are produced at low voltage; (ii) continuous and uncertain power production are clarified by variable load cycles; and (iii) to operate at the highest efficient and sustainable level, cell voltage should be controlled. In a recent study, some workers revealed that a sensor buoy could maintain power generation by a BMFC from the positioning and improvement of Benthic Unattended Generators or BUGs. Shantaram et al. [102] had the potential power to create wireless biosensor and telemetry systems. However, all of them are systems employed for a sacrificial anode that provides greater voltage, which allowed the application for the power management of economic electronics (many BMFCs provide low voltages that specify the value for the power management of economic electronics). In a more recent work, the anode of BMFCs, without scarifying the improvement of power management, enabled power for utilization to satisfy charge capacity consumed in the capacitor, but a comparison of a voltage DC-DC converter provides a voltage of 3.3 V, a sufficient voltage for a reliable device [103].

## 7. Conclusions

The proposed method of BTX pollutant removal demonstrated by BMFCs is encouraging for in situ bioremediation by microbes. These BMFCs will open new possibilities for sustainable, cost-effective and controllable ways to generate power and bioremediate BTX pollutants. The challenges of BMFCs will be conquered jointly by the efforts of scientists from many fields, such as environment, biotechnology, electrochemistry, electrical, biology and material science. From how MFCs perform bioremediation, it is evident that all of these BTX pollutants originate from industrial wastewater, which then becomes contaminated. Therefore, in the upcoming future, this will be an excellent approach for the bioremediation of BTX pollutants, and another beauty of the bioremediation of BTX pollutants is that it will also produce renewable energy through BMFC technology. 

## Figures and Tables

**Table 1:** Summary of numerous studies on BMFCs for removal of BTX with production of energy.

S. N.	Electrode Materials		Target Pollutants	Inoculum Medium	Removal Efficiency	Operation Time (days)	pH	Temperature (°C)	Power Density (mW/m^2^)	References
	Anode	Cathode								
1.	Carbon felt	Carbon felt	Benzene	Wastewater	81.6%	4	7.0	30	12.7	[82]
2.	Carbon felt	Carbon felt	Benzene	Wastewater	80%	-	-	28–30	0.0205	[83]
3.	Carbon brush	Carbon brush	Benzene	Wastewater	95%	195	-	-	38	[84]
4.	Carbon cloth	Carbon cloth	Benzene	Wastewater	80%	770	6.9–7.0	12–16	-	[85]
5.	Carbon felt	Carbon felt	Benzene	Wastewater	80%	160	7.5 ± 0.3	10–12	316	[45]
6.	Carbon brush	Carbon felt	Benzene	Minimal medium	97.10%	60	-	40	1.06	[45]
7.	Carbon rod	Carbon rod	Benzene	Wastewater	90%	120	-	-	32	[86]
8.	Carbon cloth	Carbon cloth	Toluene	xenobiotics-contaminated wastewater	96%	5	7.0	28	4.69	[87]
9.	Carbon felt	Carbon felt	Toluene	Wastewater sludge	88%	10	7.0	30	18.3	[88]
10.	Carbon brush	Carbon brush	Toluene	Groundwater	76%	45	-	30	103	[89]
11.	Carbon plate	Carbon plate	Toluene	Wastewater	-	3	6.0	30	2.6	[90]
12.	Carbon felt	Carbon felt	Toluene	-	88	-	7.0	30	18.3	[88]
13.	Carbon paper	Carbon paper	Toluene	Pyocyanin Wastewater	96	5	7.0	80	21.76	[75]
14.	Carbon sheet	Carbon sheet	Toluene	Groundwater	67.2 ± 5.7%	165	7.0	20 ± 20.5	0.001	[91]
15.	Carbon cloth	Carbon cloth	Toluene	Groundwater	91.2 ± 2.4%	-	-	-	6.19 ± 0.45	[92]
16.	Carbon rod	Carbon rod	Toluene	Coke slurry mixture	99%	-		-	25.14	[93]
17.	Carbon felt	Carbon felt	Xylene	Volatile organic compounds	35–76%	36	-	30 ± 1 °C	92.5	[94]
18.	Carbon paper	Carbon paper	Xylene	Wastewater	60.3%	-	-	-	-	[95]
19.	Reticulated carbon paper	Reticulated carbon paper	Xylene	Wastewater	61%	-	-	-	-	[96]
20.	Carbon felt	Carbon felt	Xylene	Wastewater	90%	-	-	-	-	[97]
21.	Graphite plates	Graphite plates	Xylene	Wastewater	7 ± 4 mg/L	0.34 ± 0.09	-	-	220 mA/m^2^	[98]

## Data Availability

Not applicable.
